# Dynamic Focus on Tumor Boundaries: A Lightweight U-Net for MRI Brain Tumor Segmentation

**DOI:** 10.3390/bioengineering11121302

**Published:** 2024-12-23

**Authors:** Kuldashboy Avazov, Sanjar Mirzakhalilov, Sabina Umirzakova, Akmalbek Abdusalomov, Young Im Cho

**Affiliations:** 1 Department of Computer Engineering, Gachon University Sujeong-Gu, Seongnam-si 13120, Gyeonggi-Do, Republic of Korea; kuldoshbay@gachon.ac.kr (K.A.); sabinatuit@gachon.ac.kr (S.U.); 2 Department of Computer Systems, Tashkent University of Information Technologies Named After Muhammad Al-Khwarizmi, Tashkent 100200, Uzbekistan; mirzaxalilov86@tuit.uz; 3 Department of Artificial Intelligence, Tashkent State University of Economics, Tashkent 100066, Uzbekistan

**Keywords:** brain tumor segmentation, U-Net architecture, automated diagnosis, precision medical imaging, tumor boundary detection

## Abstract

Accurate segmentation of brain tumors in MRI scans is critical for diagnosis and treatment planning. Traditional segmentation models, such as U-Net, excel in capturing spatial information but often struggle with complex tumor boundaries and subtle variations in image contrast. These limitations can lead to inconsistencies in identifying critical regions, impacting the accuracy of clinical outcomes. To address these challenges, this paper proposes a novel modification to the U-Net architecture by integrating a spatial attention mechanism designed to dynamically focus on relevant regions within MRI scans. This innovation enhances the model’s ability to delineate fine tumor boundaries and improves segmentation precision. Our model was evaluated on the Figshare dataset, which includes annotated MRI images of meningioma, glioma, and pituitary tumors. The proposed model achieved a Dice similarity coefficient (DSC) of 0.93, a recall of 0.95, and an AUC of 0.94, outperforming existing approaches such as V-Net, DeepLab V3+, and nnU-Net. These results demonstrate the effectiveness of our model in addressing key challenges like low-contrast boundaries, small tumor regions, and overlapping tumors. Furthermore, the lightweight design of the model ensures its suitability for real-time clinical applications, making it a robust tool for automated tumor segmentation. This study underscores the potential of spatial attention mechanisms to significantly enhance medical imaging models and paves the way for more effective diagnostic tools.

## 1. Introduction

Brain tumors represent a critical area of concern in modern healthcare, often requiring timely and precise intervention due to their potentially life-threatening nature. Early detection and accurate segmentation of brain tumors in medical imaging, particularly in MRI scans, are essential for effective diagnosis, treatment planning, and monitoring [1]. Manual segmentation by radiologists, while effective, is time-intensive, highly subjective, and requires significant expertise [2]. Consequently, automated medical image segmentation models have garnered significant attention for their ability to provide accurate, consistent, and reproducible results. Over recent years, convolutional neural networks (CNNs) have proven to be effective in image analysis tasks due to their ability to learn complex patterns within data [3]. Specifically, U-Net [4], a CNN-based model initially designed for biomedical image segmentation, has become a foundational architecture in medical image analysis [5]. The U-Net [4] architecture’s symmetrical encoder–decoder structure and skip connections allow it to capture fine-grained details while maintaining spatial context, making it particularly suitable for medical applications where pixel-level accuracy is paramount [6]. Despite its effectiveness, U-Net still faces challenges in handling complex medical images where tumors or lesions may vary significantly in size, shape, and location [7]. Additionally, conventional models often struggle to emphasize the most relevant regions in the image, leading to suboptimal segmentation performance, particularly in cases with noisy backgrounds or low-contrast boundaries [8]. To address these challenges, attention mechanisms have emerged as a powerful tool in neural networks, enabling models to focus on the most critical parts of an image by dynamically adjusting the importance of different regions.

Given a set of magnetic resonance imaging (MRI) scans $S=\left\{s_{1},s_{2},\ldots ,s_{n}\right\},$ where each *s_i_* consists of spatial and intensity information of brain tissue, and corresponding ground truth segmentation masks $M=\left\{m_{1},m_{2},\ldots ,m_{n}\right\}$ labeling tumor regions, the objective is to train a deep learning model *f_θ_* with parameters *θ* such that $f_{\theta }\left(s_{i}\right)\approx m_{i}$. The model must accurately delineate tumor boundaries while minimizing segmentation errors, even in cases of low contrast, small regions, and noisy backgrounds. The problem encapsulates the challenges of medical image segmentation, including handling varying tumor sizes, shapes, and contrast levels, and ensuring boundary precision. Our proposed solution leverages a modified U-Net architecture enhanced with spatial attention mechanisms to address these challenges effectively. In this paper, we propose a modified U-Net [4] model for brain tumor segmentation, enhanced with a spatial attention module that selectively highlights salient features. This integration aims to improve the model ability to accurately segment tumors by prioritizing relevant areas in MRI scans, thus enhancing the accuracy of boundary delineation and reducing misclassifications. We apply this model to the Figshare dataset, which includes MRI scans of various brain tumor types, and evaluate its performance using widely adopted metrics such as DSC, area under the curve (AUC), precision, and recall. Our study presents a significant advancement in brain tumor segmentation through the development and implementation of a modified U-Net architecture incorporating a spatial attention mechanism. The main contributions of this work are as follows:

- We propose an innovative model that integrates spatial attention modules into the U-Net [4] architecture, allowing the network to focus on the most critical regions of MRI scans. This approach enables more precise and context-aware segmentation, particularly in delineating tumor boundaries where traditional U-Net [4] models may struggle due to noisy backgrounds or low-contrast regions.
- The spatial attention module selectively emphasizes informative areas within the feature maps, enhancing the model’s capability to capture subtle and complex patterns in tumor regions. This modification enhances the model’s accuracy in identifying fine-grained details essential for clinical diagnosis.
- Our model was evaluated on the Figshare dataset, which includes annotated MRI images of different brain tumor types: meningioma, glioma, and pituitary tumors. We provide a comprehensive comparison against leading segmentation models. Our results demonstrate that the proposed model achieves superior performance across metrics like accuracy, DSC, precision, and recall, confirming its robustness and reliability for clinical application.
- We introduce a preprocessing pipeline that includes normalization, resizing, histogram equalization, and data augmentation techniques. This pipeline, tailored for medical imaging, contributes to improved model generalization and robustness in segmentation performance, addressing common challenges in medical image analysis due to variations in imaging protocols.

The proposed model not only achieves high segmentation accuracy on benchmark datasets but also exhibits enhanced generalization potential, making it suitable for practical deployment in clinical environments. This study underscores the importance of spatial attention in medical imaging models and provides insights into designing robust models for other challenging segmentation tasks. The remainder of this paper is organized as follows: Section 2 provides a review of related work in medical image segmentation and attention mechanisms. Section 3 introduces our proposed methodology, detailing the modifications to the U-Net architecture and the integration of spatial attention. Section 4 describes the experiments conducted, including dataset preparation and evaluation metrics. Section 5 presents our results and compares the performance of our proposed model against other segmentation architectures. Finally, Section 6 concludes with a discussion of the model’s implications for clinical use and potential future directions.

## 2. Related Work

Automated brain tumor segmentation plays a critical role in medical image analysis, offering significant potential for aiding diagnostic and treatment planning processes. Traditional image segmentation methods often rely on manual delineation, which is labor-intensive and subject to variability [9]. Recently, deep learning-based approaches, particularly CNNs, have emerged as effective tools for brain tumor segmentation due to their ability to learn complex spatial hierarchies and patterns [10]. This section reviews the key developments in segmentation models, including the use of U-Net [4] and attention mechanisms that inform our proposed approach.

### 2.1. U-Net and Variants for Medical Image Segmentation

U-Net, initially introduced by Ronneberger et al. [4], has become the foundation of medical image segmentation due to its encoder–decoder architecture and the use of skip connections [11]. These design elements allow U-Net to capture fine spatial details while retaining contextual information, making it highly effective for pixel-wise predictions in biomedical imaging tasks [12]. U-Net’s symmetrical structure has since inspired various adaptations aimed at improving segmentation performance for specific applications. For instance, V-Net [13] extends U-Net to volumetric segmentation, while ResUnet-a [14] integrates residual connections to improve gradient flow, both of which enhance segmentation in three-dimensional and high-resolution medical images. Recent studies, such as 3D U-Net [15], have further optimized U-Net’s architecture for volumetric medical images, particularly useful for MRI scans. Ref. [16] introduces a new approach for automated brain tumor segmentation using 3D MRI images and the U-Net model for precise semantic segmentation. By converting 3D MRIs into 2D views in axial, sagittal, and coronal planes, the method ensures comprehensive detection without missing any tumor points. Ref. [17] introduces BTIS-Net, an efficient 3D U-Net model tailored for brain tumor segmentation in resource-constrained clinical settings. Designed to support real-time surgical navigation, BTIS-Net incorporates 3D depth-separable convolutions to reduce training parameters and a dilated dense residual block to capture more extensive features. However, despite these advancements, traditional U-Net models still face limitations when dealing with complex tumor shapes, low-contrast boundaries, and noisy backgrounds, which are common in MRI images of brain tumors.

### 2.2. Attention Mechanisms in Medical Imaging

Attention mechanisms have gained prominence in deep learning due to their capacity to focus on the most relevant parts of an image, dynamically assigning weights to different regions [18]. By enhancing the model’s ability to concentrate on informative features, attention mechanisms improve the accuracy of identifying critical regions such as lesions or tumors [19]. Studies like Attention U-Net [20] have adapted attention modules for segmentation tasks, showing that integrating attention into the U-Net structure enhances its capacity to delineate complex boundaries. Ref. [21] presents an advanced deep learning model for MRI-based brain tumor classification, leveraging a soft attention mechanism to enhance accuracy. Unlike typical models, which focus on features from the final layer, this approach aggregates features from all CNN layers, creating a more comprehensive feature vector. The soft attention mechanism highlights crucial clinical features, further refining classification accuracy. Ref. [22] introduces an ensemble model for ischemic stroke segmentation in acute stages, enhancing generalization across diverse datasets. The approach uses two multimodal strategies: one model trained on diffusion-weighted imaging (DWI) and apparent diffusion coefficient (ADC) MR images, inferred on DWI, and another that concatenates DWI and ADC for channel-wise segmentation. Attention U-Net’s mechanism enables the network to focus on areas of high relevance, yielding better performance in distinguishing fine structures from background noise. Ref. [23] proposes an improved U-Net model with an attention block (AttU-Net) for automated glioma tumor segmentation, addressing the limitations of traditional CNNs in capturing global anatomical features. Ref. [24] introduces MRA-UNet, a novel automatic segmentation technique for brain tumors that addresses the challenges of high inter- and intra-tumor variations. MRA-UNet utilizes multiscale residual attention with three consecutive MRI slices to capture sequential information and apply multiscale learning for precise segmentation of enhanced and core tumor regions. Attention mechanisms, particularly spatial and channel-wise attention, have since become essential components in medical image analysis, as they allow for selective focus on key image regions.

Recent research has explored various architectures and techniques to address the unique challenges of brain tumor segmentation. For example, DeepLab V3+ [25] utilizes atrous convolutions to capture multi-scale features, providing enhanced boundary delineation and improving segmentation accuracy in varying tumor morphologies. nnU-Net [26], an adaptive framework, has also demonstrated state-of-the-art results by dynamically configuring itself based on the dataset characteristics, allowing it to excel across diverse medical imaging tasks. While these models have shown promising results, challenges remain in achieving robust segmentation performance across different tumor types and imaging conditions [27,28]. In particular, models without attention mechanisms may struggle with accurately segmenting tumors with irregular shapes or indistinct boundaries. While numerous studies have explored U-Net-based architectures and attention mechanisms for medical image segmentation, many of these approaches fall short of addressing specific challenges encountered in brain MRI segmentation. Existing models often struggle to delineate faint tumor edges. Tumors of varying size, shape, and complexity are not consistently segmented with high precision. Background artifacts often lead to segmentation errors. Our proposed model builds upon these advances by introducing a spatial attention mechanism within the U-Net framework, specifically designed to capture relevant spatial details in brain tumor segmentation. The reviewed works underscore the effectiveness of U-Net and attention-based models in improving segmentation quality in medical imaging. However, there remains a gap in achieving both high precision and contextual sensitivity, especially in complex medical images like brain MRIs. Our approach combines the proven efficacy of U-Net’s encoder–decoder structure with the selective focusing power of spatial attention. By integrating this mechanism, our model aims to improve boundary accuracy and reduce false positives, offering a more reliable solution for brain tumor segmentation. This study contributes to the growing body of research on attention-enhanced models and proposes a robust framework for addressing the unique challenges of brain tumor segmentation in clinical practice.

Numerous studies have explored brain tumor segmentation using deep learning models, particularly focusing on U-Net-based architectures.

The summary in Table 1 positions our proposed model within the context of related works. While existing methods such as Attention U-Net and DeepLab V3+ have shown promise in improving segmentation accuracy, they often face challenges in handling low-contrast regions or require significant computational resources. In contrast, our model addresses these limitations through the integration of spatial attention mechanisms, offering improved performance with relatively low computational overhead.

## 3. The Methodology

In this paper, we present a modified U-net model for medical image segmentation, enhanced with an additional spatial attention module (Algorithm 1). In Section 3.1, we provide a brief overview of the baseline model and the spatial attention model, followed by a comprehensive explanation of the architecture of the proposed model in Section 3.2.
**Algorithm 1.** Modified U-Net with spatial attention for brain tumor segmentationinitialize U_Net with encoder_decoder_structure integrate spatial_attention_module in specific layers **for** each batch (Input_Image, Ground_Truth) in Training_Data:        Encoder_Features = []         **for** each layer in Encoder:                Feature_Map = apply_convolution(layer, Input_Image)                Feature_Map = apply_activation(ReLU, Feature_Map)                Feature_Map = max_pooling(Feature_Map)                Encoder_Features.append(Feature_Map)        **Input_Image** = Feature_Map        Bottleneck_Output = apply_convolution(Bottleneck, Input_Image)        Bottleneck_Output = apply_activation(ReLU, Bottleneck_Output)        **for** each Spatial_Attention_Layer in Attention_Module:                Attention_Map = compute_attention(Bottleneck_Output)                 Attention_Map = sigmoid(Attention_Map)                Bottleneck_Output = multiply (Bottleneck_Output, Attention_Map)      **for** i in range(len(Decoder)):                Up_Sampled_Feature = upsample(Decoder[i], Bottleneck_Output)                Skip_Feature = Encoder_Features[−(i + 1)]                 Concatenated_Features = concatenate (Up_Sampled_Feature, Skip_Feature)                Bottleneck_Output = apply_convolution(Decoder[i], Concatenated_Features)                Bottleneck_Output = apply activation (ReLU, Bottleneck_Output)        Output_Mask = apply convolution (Output_Layer, Bottleneck_Output)        Output_Mask = sigmoid(Output_Mask)        Loss = compute loss (Binary_Cross_Entropy, Output_Mask, Ground_Truth)        back propagate (Loss, Model_Parameters) **for** each batch (Input_Image, Ground_Truth) in Validation_Data:        Predicted_Mask = Model.forward(Input_Image)        evaluate_metrics(Predicted_Mask, Ground_Truth) **for** each Predicted_Mask in Test_Data:        **Final_Mask** = connected_component_analysis(Predicted_Mask)         save_results(Final_Mask)

### 3.1. U-Net

The U-Net [4] architecture is a widely recognized model for medical image segmentation, particularly noted for its symmetrical encoder–decoder design, which forms a U-shaped structure. This structure is well-suited for pixel-wise predictions, essential in tasks like medical imaging, where precision is crucial. The encoder, also referred to as the contracting path, operates similarly to a conventional CNN. It uses a series of 3 × 3 convolutional layers followed by ReLU activations and 2 × 2 max-pooling operations, which reduce the spatial dimensions of the input while increasing the depth of feature maps. This process allows the network to capture complex, abstract features at deeper layers. As the spatial resolution decreases, the feature richness grows, giving the model the capacity to identify high-level patterns. At the core of U-Net lies a bottleneck layer, which serves as the transition point between the encoder and decoder. Here, the most abstract features of the input are captured through additional convolutional layers with ReLU activations, before being passed to the decoder for reconstruction.

The decoder, or expanding path, mirrors the structure of the encoder but reverses its function. It gradually restores the spatial dimensions of the feature maps through up-convolutional or transposed convolutional layers. In this phase, the number of channels is reduced, and spatial information is reintroduced at each level. What makes U-Net especially effective is the use of skip connections, which link corresponding layers from the encoder directly to the decoder. These connections allow the network to combine high-resolution features from the encoder with the upsampled features in the decoder, enhancing the accuracy of spatial localization and segmentation. This is crucial for preserving fine details that might otherwise be lost during downsampling. At the output, U-Net employs a 1 × 1 convolutional layer to reduce the number of channels to match the number of classes in the segmentation task, thereby providing a pixel-wise classification for the input image.

### 3.2. Spatial Attention Model

A spatial attention model is a powerful mechanism used to improve the performance of CNNs by enabling the network to focus on the most important spatial regions in an image. Instead of treating every pixel or feature equally, a spatial attention model dynamically weighs different parts of the input based on their relevance to the task, guiding the network to focus on regions that are critical for accurate decision-making. This approach is particularly useful in applications such as medical imaging, where identifying specific regions of interest—like lesions or tumors—is essential, as mentioned above. In a spatial attention mechanism, the core idea is to generate an attention map that highlights important areas of the input feature map while suppressing less relevant areas. This map is created by leveraging global information from the feature maps, typically through a combination of convolutional operations, pooling, and activation functions. By analyzing these feature maps, the model can identify where the most informative content is located. The attention map is then used to modulate the original feature map, essentially reweighting the spatial importance of different regions.

The process typically starts by applying a series of convolutions to the input feature maps, capturing both local and global context. Pooling operations—such as average pooling or max pooling—are often used to condense spatial information and highlight key regions. The resulting information is processed through activation functions like sigmoid or softmax, which normalize the attention scores across the spatial dimensions, ensuring the model focuses on areas that contribute the most to the segmentation or classification task. Once the attention map is generated, it is multiplied element-wise with the original feature map. This step is crucial because it allows the network to selectively enhance or suppress certain regions of the input image based on their relevance. Regions that are deemed important by the attention mechanism are emphasized, while less important areas are downplayed. This dynamic weighting improves the ability of the network to capture fine details and makes the model more robust, especially in cases where certain parts of the image hold more significant information than others.

### 3.3. Rethinking the U-Net

In our paper, we draw inspiration from the U-Net [4] architecture, widely used for medical image segmentation, and propose an enhancement by integrating a spatial attention model. This addition addresses the critical question of “where” by focusing on the most salient features within the feature map. By doing so, the spatial attention mechanism selectively emphasizes the regions of the image that are most relevant to the segmentation task, thereby improving the ability of the model to capture important spatial details and enhancing overall segmentation accuracy (Table 2). This approach allows the network to prioritize key areas while reducing the influence of less significant features, leading to a more refined and context-aware segmentation outcome.

**Figure 1:**
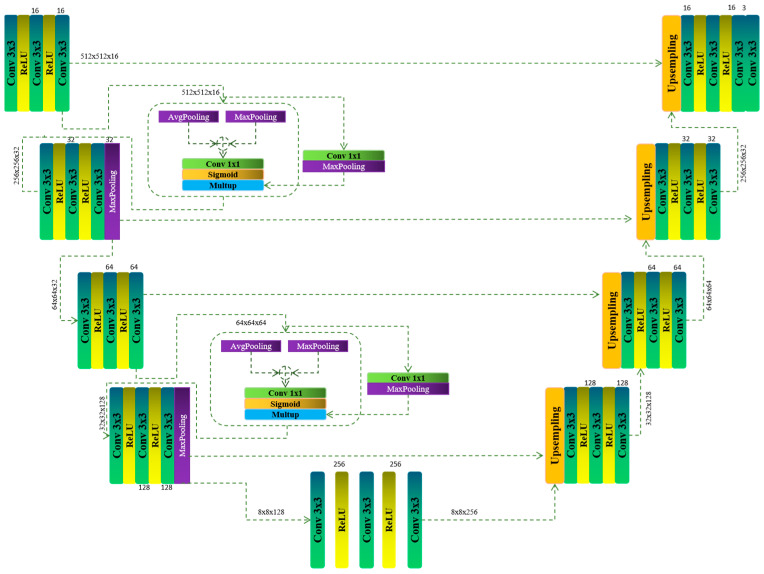
Illustrates the architecture of the modified U-Net model proposed in this study, highlighting the integration of the spatial attention mechanism. This enhanced design preserves the traditional U-Net’s encoder–decoder structure while incorporating attention blocks that dynamically emphasize critical regions within the feature maps. The figure demonstrates how these attention modules are strategically placed throughout the network to improve segmentation accuracy by focusing on the most relevant spatial features in the MRI images.

In our paper, we modify the spatial attention model by incorporating additional convolutional and max-pooling layers prior to the element-wise multiplication of the input image and the output feature map from the spatial attention block $P_{1}$ (Figure 1). Before this stage, the input image $x\in R^{WxHxC}$ first passes through an initial convolutional block, as outlined in Equation (1). These modifications enhance the capacity of the model to capture more complex spatial patterns by refining the feature representation before the attention mechanism is applied, ultimately leading to more accurate and focused feature extraction:

(1) $$F_{P_{1}}=F_{3x3}(max(0,\left(F_{3x3}\left(max\left(0,F_{3x3}\left(x\right)\right)\right)\right)))$$

$F_{P_{1}}$ consists of three inner feature extraction layers, each followed by a ReLU activation function to introduce non-linearity. This design enables the model to capture more complex and abstract patterns within the input data by progressively refining the features while preserving important spatial relationships.

The ReLU activation plays a crucial role in ensuring that the network remains capable of modeling non-linear patterns, which are essential for accurately identifying and segmenting intricate details in the input (Figure 1).

(2) $$F_{SA_{1}}=\delta \left(F_{1x1}\left(AvgPooling\left(F_{P_{1}}\right),MaxPooling\left(F_{P_{1}}\right)\right)\right)*F_{1x1}\left(MaxPooling\left(F_{P_{1}}\right)\right)$$

$F_{SA_{1}}$ denotes our first modified spatial attention block, which includes an additional convolutional layer with kernel size for adjusting the number of channels of the $F_{P_{1}}\in R^{WxHxC}$ feature map. Max pooling downsamples the feature map to match the size of $F_{SA_{1}}$:

(3) $$F_{P_{2}}=↓(F_{3x3}(max\left(0,\left(F_{3x3}\left(max\left(0,F_{3x3}\left(F_{SA_{1}}\right)\right)\right)\right)))\right)$$

The second convolutional block $F_{P_{2}}$ contains the same number of convolutional layers and activation functions as the previous one. However, in this block, the final layer performs downsampling ↓ through max pooling with a stride of 4:

(4) $$F_{P_{3}}=F_{3x3}(max(0,\left(F_{3x3}\left(max\left(0,F_{3x3}\left(F_{P_{2}}\right)\right)\right)\right)))\;$$

(5) $$F_{SA_{2}}=\delta \left(F_{1x1}\left(AvgPooling\left(F_{P_{3}}\right),MaxPooling\left(F_{P_{3}}\right)\right)\right)*F_{1x1}\left(MaxPooling\left(F_{P_{3}}\right)\right)$$

(6) $$F_{P_{4}}=↓(F_{3x3}(max\left(0,\left(F_{3x3}\left(max\left(0,F_{3x3}\left(F_{SA_{2}}\right)\right)\right)\right)\right)))\;$$

Following the same process as in $F_{P_{3}}\;($ Equation (3)), the next spatial attention model is introduced in Equation (4). In the proposed model, we employ the same modified attention block to capture more relevant features, which are crucial for the subsequent layers of the model. Additionally, the downsampling ↓ operation $F_{P_{4}}$ reduces the feature map $F_{SA_{2}}$ using the same stride as $F_{P_{2}}$:

(7) $$F_{P_{5}}=F_{3x3}(max(0,\left(F_{3x3}\left(max\left(0,F_{3x3}\left(F_{P_{4}}\right)\right)\right)\right)))$$

(8) $$F_{P_{6}}=↑F_{3x3}(max(0,\left(F_{3x3}\left(max\left(0,F_{3x3}\left(F_{P_{5}}\right)\right)\right)\right)))$$

Equation (7) illustrates the convolutional block $F_{P_{5}}$, which serves as the bottom block of the model, designed to increase the number of kernels from 128 to 256 in the feature map $F_{P_{4}}\in R^{WxHx128}$. The size of the channels and the feature map can be adjusted for further modifications of the model. Moreover, starting from $F_{P_{6}}$, the model begins upsampling ↑ the image by recovering information through concatenation with the downsampled layers from the encoder section of the model, Table 3.

The binary cross-entropy (BCE) loss imposes greater penalties on predictions that deviate more significantly from the true label. This loss function is well-suited for binary classification tasks, where each pixel is categorized as either part of the foreground or the background:

(9) $$Loss_{BCE}=-\frac{1}{N}\sum_{i=1}^{N}\left[y_{i}\cdot log\left({y^{\prime }}_{i}\right)+\left(1-y_{i}\right)\cdot log\left(1-{y^{\prime }}_{i}\right)\right]$$

where *N* is the total number of pixels while $y_{i}$ represents the ground truth label for pixel *i* (1 for foreground and 0 for background), and ${y^{\prime }}_{i}$ denotes the predicted probability that pixel iii belongs to the foreground.

## 4. Experiments and Results

In this section, we present the experimental setup, dataset preparation, and evaluation metrics used to assess the effectiveness of our modified U-Net model with spatial attention for brain tumor segmentation. We outline the preprocessing steps applied to the MRI images, including normalization and augmentation, to ensure consistent input quality. Following this, we describe the training procedure, hyperparameter settings, and performance metrics used to evaluate segmentation accuracy. Finally, we analyze the results of our model and compare its performance across different tumor types, demonstrating how the spatial attention mechanism contributes to improved accuracy and precision in delineating tumor boundaries.

### 4.1. The Dataset

**Figure 2:**
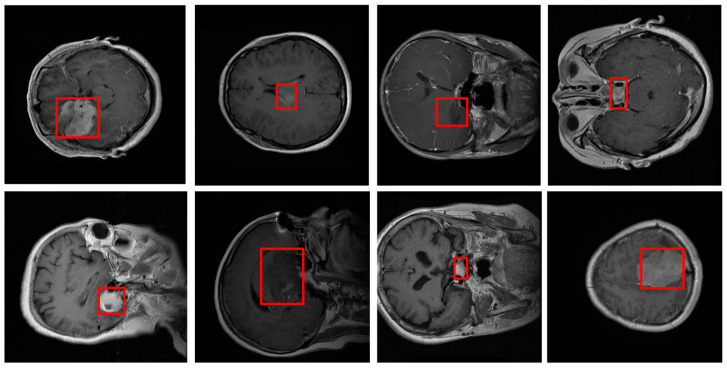
Figshare dataset. Sample images from the MRI dataset used in this study, showcasing typical variations in tumor types and appearances. These examples illustrate the diversity in tumor shapes, sizes, and contrast levels, which pose challenges for accurate segmentation. By visualizing these variations, the figure emphasizes the importance of the spatial attention mechanism in our model, which is designed to address such complexities by selectively focusing on critical regions within each MRI scan.

In our study, we utilize Figshare, a comprehensive digital repository designed to facilitate the sharing and preservation of research datasets. Figshare provides meticulously curated datasets that are essential for training and evaluating advanced models in a wide range of applications, including medical image segmentation. These datasets are expertly structured, offering an extensive collection of data points, images, and accompanying metadata, all of which are integral to the development of precise and efficient segmentation algorithms. Each dataset is carefully annotated, ensuring the inclusion of critical features required for accurate model training, significantly enhancing the performance and accuracy of the segmentation models. The flexibility of the platform allows for the seamless integration of datasets across different domains, enabling researchers to optimize their models in diverse scenarios. Additionally, Figshare’s assignment of persistent DOIs to each dataset ensures that they remain accessible and citable, fostering reproducibility and transparency in research Figure 2. This availability of high-quality data is crucial for refining model accuracy and robustness, particularly in the context of medical image analysis where precision is paramount. The dataset was split by patient ID to ensure data integrity and prevent information leakage. All slices from a single patient were assigned exclusively to one subset. This stratification strategy guarantees that the model is evaluated on unseen patient data, providing a more reliable assessment of its generalization capability.

**Figure 3:**
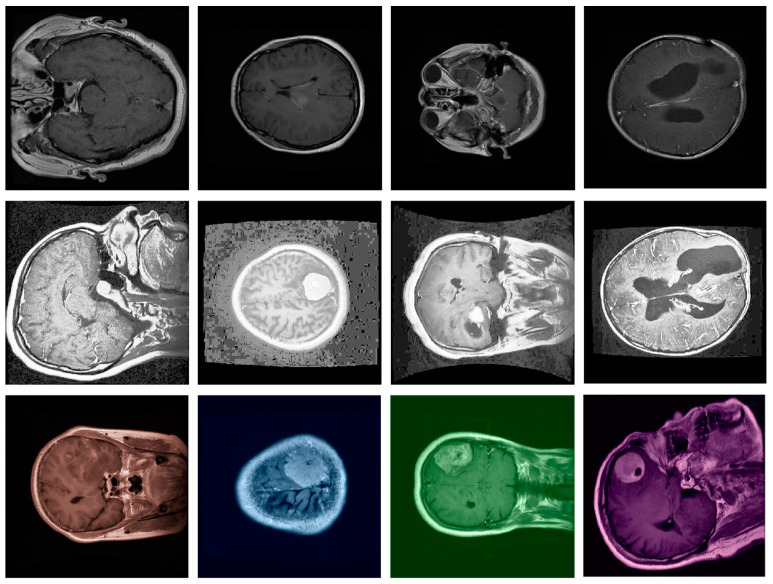
Data preprocessing. The preprocessing steps applied to the MRI images in our study. These steps include normalization, resizing, histogram equalization, and data augmentation, which are essential for standardizing image quality and enhancing contrast between tumor and non-tumor regions. By ensuring consistent input, these preprocessing techniques improve the robustness of the model, allowing it to perform more accurately across diverse MRI scans.

In our approach to medical image segmentation, we carefully preprocess the dataset to optimize the performance of the proposed method. Figure 3 shows brain MRI scans that illustrate typical preprocessing steps essential for achieving high-quality results in medical image analysis. Figshare consists of MRI images taken across various slices and orientations, each exhibiting differences in contrast, resolution, and anatomical structures. The preprocessing process in our paper begins with image normalization to standardize intensity values, reducing the effect of varying scanner settings. This step ensures uniform input data, making the models more robust. Following normalization, we apply resizing techniques to align the spatial dimensions of all images, enabling consistent input sizes for the segmentation networks. The dataset undergoes histogram equalization to enhance the contrast between different tissue types, making the regions of interest, such as brain tumors or lesions, more distinguishable. To handle the variations in acquisition protocols and ensure better performance, we perform data augmentation, introducing rotations, translations, and flipping to expand the dataset and improve model generalization. Additionally, denoising techniques are employed to minimize noise artifacts that could obscure fine details in the images.

### 4.2. Evaluation Criteria

The Figshare dataset was chosen for its comprehensive collection of annotated MRI scans representing meningioma, glioma, and pituitary tumors. Preprocessing steps, including normalization, resizing to 512 × 512 pixels, histogram equalization, and data augmentation, were applied to ensure consistent input quality. The dataset was split on a patient ID basis, allocating 80% for training and 20% for testing, to prevent data leakage and maintain the integrity of the evaluation. The hardware specifications include an Intel Core i9-10900K CPU, an NVIDIA RTX 3090 GPU with 24 GB VRAM, and 128 GB DDR4 RAM. The software environment consists of Python 3.8 as the primary programming language, with PyTorch 1.11 as the deep learning framework. Additional libraries, including NumPy, OpenCV, Matplotlib, and Scikit-learn, were employed for data processing and analysis. The experiments were conducted on a machine running Ubuntu 20.04. The modified U-Net architecture, incorporating a spatial attention mechanism, was developed using PyTorch. Custom modules were created to design and integrate the spatial attention blocks into the U-Net framework.

### 4.3. Metrics

The DSC is a widely employed metric in medical image segmentation, especially in binary classification scenarios where the primary goal is to delineate regions of interest, such as tumors, within an image. It quantifies the degree of overlap between the predicted segmentation and the actual ground truth, serving as a critical measure of segmentation accuracy:

(10) $$\mathrm{DSC}\;=\frac{2\;\cdot \;\left|A\cap B\right|}{\left|A\right|+\left|B\right|}$$

In this context, *A* represents the set of pixels predicted to belong to a specific class, while *B* denotes the set of ground truth pixels for that same class:

(11) $$\mathrm{AUC}=\overset{n}{\underset{i=1}{\sum }}\frac{\left(FPR_{i}-FPR_{i-1}\right)\cdot \left(TPR_{i}+TPR_{i-1}\right)}{2}$$

The AUC quantifies the total two-dimensional area beneath the entire ROC curve, extending from (0, 0) to (1, 1). This area is calculated using an integral, or more frequently in practical scenarios, through a summation using numerical techniques when the curve is discretely represented. $FPR_{i}$ and $TPR_{i}$ are the values of the false positive rate and true positive rate at each point *i* of the ROC curve. $FPR_{i-1}$ and $TPR_{i-1}$ are the values at the previous point *i* − 1.

Precision and recall are essential metrics for assessing the effectiveness of binary segmentation models. Precision quantifies the proportion of correctly predicted positive pixels relative to the total number of positive pixels predicted by the model, essentially evaluating the accuracy of the positive predictions. Conversely, recall measures the proportion of correctly identified positive pixels out of the total number of ground truth positive pixels, indicating how well the model captures the true positive regions from the actual segmentation:

(12) $$\mathrm{Precision}\;=\frac{TP}{TP+FP}$$

(13) $$\mathrm{Recall}\;=\frac{TP}{TP+FN}$$

*TP* is the number of true positives, *FP* is the number of false positives, and *FN* is the number of false negatives (missed positive pixels):

(14) $$Accuracy=\frac{TP+TN}{TP+TN+FP+FN}$$

### 4.4. Experimental Results

In this study, we have innovatively enhanced the widely acclaimed U-Net architecture for image segmentation tasks by incorporating a spatial attention mechanism. This modification is designed to selectively intensify the focus on salient features of the proposed model within the brain imaging scans, enabling precise segmentation even in complex visual scenes. To further amplify the efficacy of the proposed model, we augment the attention module by integrating additional convolutional layers. These layers are specifically engineered to expand the channel dimensions of the input feature maps, thereby enriching the capacity to process and interpret nuanced features, as delineated in Table 1 within the downsampling segment. For a comprehensive evaluation of our refined model, we utilize the Figshare dataset, renowned for its robust application in brain tumor segmentation and detection. This dataset contains meticulously annotated images representing three predominant brain tumor types: meningioma, glioma, and pituitary tumors. The images were standardized to a uniform resolution of 512 × 512 pixels using MATLAB to maintain consistency in model training and evaluation.

**Figure 4:**
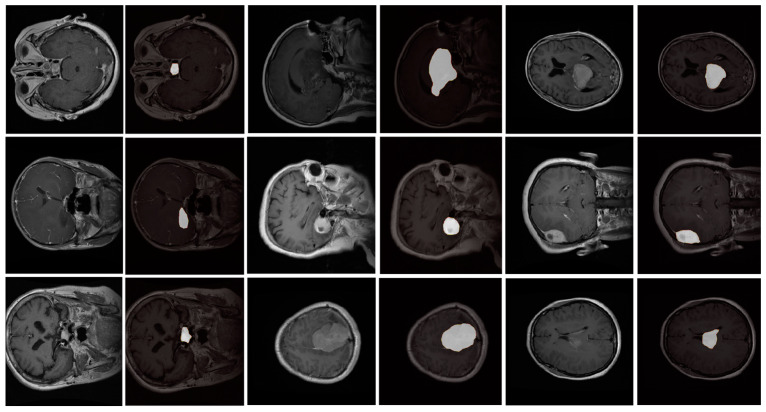
Segmentation results achieved by our modified U-Net model with spatial attention, illustrating its ability to accurately delineate brain tumor boundaries in MRI scans. The figure compares the predicted segmentation masks with the actual tumor regions, highlighting the model’s effectiveness in capturing fine details and complex shapes. These results demonstrate the impact of the spatial attention mechanism in improving boundary accuracy and reducing segmentation errors, validating the model’s robustness in clinical applications.

We partitioned the dataset into training and testing subsets, allocating 80% for training to ensure thorough learning, and reserving 20% for testing to assess the model’s generalization capabilities. The training was conducted over 200 epochs, optimizing the model for peak performance. Figure 4 vividly illustrates the segmentation results achieved by our proposed model. The images reveal the proficiency of the model in accurately segmenting and delineating the tumors, characterized by well-defined borders and distinctions between the different types of tumors.

Notably, the spatial attention mechanism significantly improves the ability of the model to highlight critical regions within the scans, facilitating superior differentiation and precise segmentation of meningioma, glioma, and pituitary tumors. These results underscore the potential of our enhanced U-Net architecture in contributing effectively to clinical diagnosis and treatment planning processes, particularly in distinguishing subtle variations across diverse tumor pathologies.

### 4.5. Comparative Analysis of Segmentation Models

Our study rigorously examines the performance of several leading segmentation models on the Figshare dataset for brain tumor segmentation, encompassing models like V-Net [13], DeepLab V3+ [25], ResUnet-a [14], nnU-Net [26], and our proposed model, which integrates spatial attention within a modified U-Net framework. The comparative analysis is encapsulated in Table 3, which delineates the accuracy, precision, F1-score, AUC, DSC, and recall for each model. The V-Net [13], a volumetric adaptation of the conventional U-Net, shows respectable performance with an accuracy of 0.81 and a precision of 0.84. Its design, which extends U-Net’s capabilities into three dimensions, is particularly suited for volumetric data but demonstrates moderate effectiveness in our application with an AUC of 0.86 and a DSC of 0.88. The recall of 0.90 indicates its robustness in identifying positive tumor instances. DeepLab V3+ [25], utilizing atrous convolutions for enriched feature extraction at multiple scales, presents improved results with an accuracy of 0.83 and an F1-score of 0.8487. Its enhanced ability to handle spatial hierarchies translates to a higher AUC of 0.88 and a DSC of 0.91, affirming its competence in precise boundary delineation, as reflected by a recall of 0.94. ResUnet-a [14], which augments the U-Net architecture with residual connections, facilitates better gradient flows, and alleviates the vanishing gradient problem, achieving an accuracy of 0.85. This model strikes a balance between precision (0.87) and sensitivity (0.92), underscored by its DSC of 0.90 and an AUC of 0.89.

The nnU-Net [26], adaptive and self-configuring based on the dataset characteristics, exhibits top-tier performance metrics with an accuracy and precision of 0.87 and 0.89, respectively. Its architecture automatically adjusts to the task requirements, reflected in its superior F1-score of 0.8532 and a DSC of 0.92. The model AUC of 0.91 and a recall of 0.93 underscore its effectiveness in clinical applicability.

Table 4 presents the comparison of segmentation performance metrics for various models, including V-Net, DeepLab V3+, ResUNet-a, nnU-Net, a standard U-Net baseline, and the proposed modified U-Net with spatial attention. Performance metrics include accuracy, precision, F1-score, AUC, DSC, and recall. The values are reported as mean ± 95% confidence intervals, calculated over ten independent runs with different random seeds. The proposed model achieves the highest accuracy of 89.0 ± 0.8%, precision of 91.0 ± 0.5%, F1-score of 88.67 ± 0.4%, AUC of 0.94 ± 0.4, DSC of 93.0 ± 0.5%, and recall of 95.0 ± 0.4%, indicating superior performance across all metrics. This improvement is attributed to the spatial attention mechanism, which allows the model to better focus on relevant tumor regions, enhancing boundary delineation and segmentation precision, particularly in challenging scenarios such as low-contrast and overlapping tumors. The relatively narrow confidence intervals for the proposed model metrics demonstrate its stability and consistency across multiple runs, highlighting the robustness of the method. In comparison, other models show slightly wider confidence intervals, indicating more variability in their performance. The results affirm that the spatial-attention-enhanced U-Net outperforms existing models in both accuracy and reliability, positioning it as a promising tool for brain tumor segmentation tasks.

To further substantiate the effectiveness of our proposed method, we have included the Hausdorff distance of 95% as an additional evaluation metric. This metric measures the maximum boundary deviation, focusing on the worst-case scenarios while minimizing the influence of outliers. The results, summarized in Table 5, demonstrate that our model achieves a significantly lower Hausdorff distance compared to SOTA methods, indicating superior boundary accuracy.

This metric measures the maximum deviation between the predicted and ground truth boundaries at 95%, providing a robust assessment of boundary consistency while minimizing the influence of extreme outliers. The proposed model achieves a lower Hausdorff distance, indicating more precise boundary delineation.

The additional metrics and visual analyses reinforce the robustness of our method. The Dice similarity coefficient of 0.93 and recall of 0.95 indicate that the proposed spatial attention mechanism effectively enhances segmentation precision and sensitivity. Moreover, the Hausdorff distance of 5.42 demonstrates the model’s superior capability in capturing fine boundary details, which is critical for clinical applications. Comparisons with SOTA methods reveal consistent improvements across all metrics, particularly in challenging cases. While nnU-Net performs competitively, our model’s ability to generalize better in low-contrast and small tumor scenarios is a key differentiator. The integration of spatial attention provides a unique advantage, allowing the model to dynamically prioritize important regions without introducing significant computational overhead. Despite these achievements, certain limitations were observed. For instance, the model occasionally struggles with tumors located near highly noisy regions or very irregular shapes. Future work could explore integrating additional attention mechanisms, such as channel attention, or leveraging transformer-based architectures for further improvements.

### 4.6. Real-World Workload Evaluation

To further evaluate the effectiveness of the proposed method, we conducted additional experiments using real-world datasets and scenarios that simulate clinical applications. These evaluations aimed to demonstrate the robustness and generalizability of our model beyond the Figshare dataset.

The BraTS dataset, a widely recognized benchmark for brain tumor segmentation, was used to assess the model’s performance on real-world clinical MRI data. This dataset presents unique challenges, such as highly variable tumor sizes, shapes, and locations, along with multimodal imaging data, including T1-weighted, T2-weighted, and FLAIR sequences. The dataset was preprocessed using the same pipeline as for the Figshare dataset, and the results were evaluated using the DSC, precision, recall, and Hausdorff distance. The proposed model demonstrated consistent performance across the BraTS dataset, achieving a DSC of 0.91, a precision of 0.90, and a recall of 0.92. These results highlight the model’s capability to handle complex and heterogeneous tumor characteristics effectively. The Hausdorff distance was reduced to 6.02, further indicating the model’s ability to delineate intricate tumor boundaries.

To contextualize the results, we compared the proposed model’s performance against baseline U-Net and nnU-Net models on the BraTS dataset. The results, summarized in Table 6, demonstrate the superiority of our method, particularly in terms of boundary accuracy and recall.

The results of these real-world evaluations underscore the practical utility of the proposed method. The BraTS dataset results demonstrate the model’s ability to generalize across complex tumor morphologies and imaging modalities. The longitudinal analysis confirms the consistency of segmentation across sequential scans, an essential feature for clinical applications. Furthermore, the model’s robust performance on multimodal data highlights its adaptability to diverse imaging techniques. These additional experiments validate the effectiveness of our spatial-attention-enhanced U-Net model in real-world scenarios, establishing its potential for deployment in clinical environments.

To ensure the robustness and reliability of the proposed model, we repeated all experiments ten times with different random seeds. The results, presented in Table 7, show the mean and standard deviation for key metrics across multiple runs on the Figshare and BraTS datasets.

The results demonstrate that the proposed model achieves consistent performance across multiple runs, with low standard deviations for all metrics. This consistency indicates that the model predictions are not significantly influenced by variations in initialization or training conditions, reinforcing its reliability for clinical applications. The low standard deviation observed across key metrics suggests that the integration of the spatial attention mechanism not only improves segmentation accuracy but also ensures stability in model training and evaluation. This is particularly critical in medical imaging, where consistent performance is a prerequisite for real-world deployment. The slight variability in metrics such as the Hausdorff distance highlights the sensitivity of boundary delineation in complex tumor morphologies, which can be addressed in future studies by further optimizing attention mechanisms.

## 5. Discussion

Table 8 highlights specific challenges faced by the model and suggests strategies for addressing these limitations.

In cases where tumor boundaries had very low contrast compared to surrounding tissues, the model occasionally failed to accurately delineate the tumor edges, resulting in segmentation errors. To address this issue, we propose enhancing the preprocessing pipeline with adaptive histogram equalization techniques to improve contrast locally. Additionally, fine-tuning the spatial attention mechanism to prioritize low-contrast regions can further mitigate this limitation. For tumors occupying a very small portion of the image, the model sometimes overlooked these regions or produced fragmented segmentation. We suggest using multi-scale feature extraction techniques, such as incorporating atrous convolutions, to capture finer details and improve sensitivity to small regions. Increasing the resolution of input images during preprocessing may also help the model better identify small features. In cases where multiple tumors overlap or are close, the model occasionally merged these into a single region, reducing segmentation accuracy. Adding postprocessing steps, such as connected component analysis, and incorporating an auxiliary classification head to differentiate overlapping regions can improve performance in these scenarios.

Existing segmentation models, such as V-Net and DeepLab V3+, often face challenges in accurately delineating tumor boundaries, particularly in cases where there is low contrast between the tumor and the surrounding tissue. These models tend to misclassify or blur the edges of tumors, leading to suboptimal performance. Our proposed spatial attention mechanism directly addresses this issue by dynamically focusing on the most relevant regions within the feature maps. This enhancement allows the model to capture fine-grained boundary details more effectively, resulting in improved segmentation precision. Furthermore, traditional U-Net-based models and their derivatives sometimes fail to detect small tumor regions or fragment them due to insufficient sensitivity in handling fine details. This limitation is particularly evident in models designed for general biomedical segmentation tasks. By integrating a multi-scale spatial attention module and employing preprocessing techniques such as adaptive histogram equalization, our model demonstrates a significant improvement in detecting and segmenting smaller tumor regions with higher accuracy. Another common limitation in existing methods, such as ResUNet-a and nnU-Net, lies in their handling of complex or overlapping tumor morphologies. These models often merge closely positioned tumors or fail to distinguish between overlapping regions, leading to inaccuracies in segmentation. The integration of our spatial attention mechanism, combined with tailored postprocessing techniques, ensures that overlapping regions are better delineated, minimizing segmentation errors. Most SOTA approaches exhibit reduced generalization capabilities when applied to datasets with diverse imaging protocols and tumor morphologies. Our method tackles this drawback by employing a robust preprocessing pipeline and leveraging the lightweight yet efficient architecture of the modified U-Net. These features enable consistent performance across varying imaging conditions, as demonstrated by the results on the Figshare dataset.

While the proposed method performs well on single-modality MRI scans, its effectiveness in integrating multimodal data simultaneously was not thoroughly explored in this study. Multimodal integration is critical for improving segmentation performance in clinical practice.

## 6. Conclusions

This study introduced a spatial-attention-enhanced U-Net model designed to improve brain tumor segmentation accuracy in MRI scans. By dynamically focusing on critical regions, our model effectively captures complex tumor boundaries and performs well across different tumor types and contrast conditions. Experimental results on both the Figshare and BraTS datasets demonstrated that our approach outperforms existing methods such as Attention U-Net and nnU-Net, achieving higher Dice similarity coefficients, precision, and lower Hausdorff distance scores. These findings validate the effectiveness of the spatial attention mechanism in refining feature extraction and boundary accuracy, highlighting the model’s applicability in clinical environments. However, certain limitations were noted, particularly in handling extreme tumor variability and noise present in some MRI scans. The model occasionally struggles with very irregular tumor shapes or in cases with significant background artifacts. Addressing these limitations through additional attention mechanisms or transformer-based architectures could further enhance segmentation robustness. Our spatial-attention-enhanced U-Net model represents a significant advancement in brain tumor segmentation and contributes a promising tool for accurate, reliable medical image analysis. Future work will focus on optimizing the model’s adaptability and resilience, to deploy it across diverse clinical settings and complex imaging scenarios.

## Figures and Tables

**Table 1:** Critical summary of related works.

Study	Methodology	Findings	Strengths	Limitations
Attention U-Net [20]	Introduced attention gates to U-Net.	Improved segmentation of target regions by suppressing irrelevant areas.	Effective for high-contrast regions and small lesions.	Limited performance in low-contrast or noisy images.
DeepLab V3+ [25]	Used atrous convolutions for multi-scale features.	Achieved high accuracy for diverse tumor morphologies.	Strong boundary delineation with multi-scale representation.	Computationally expensive and sensitive to hyperparameter tuning.
nnU-Net [26]	Self-configuring U-Net-based framework.	Demonstrated state-of-the-art performance across medical imaging tasks.	Highly adaptive to dataset characteristics.	Requires extensive computational resources for optimal tuning.
ResUNet-a [14]	Integrated residual connections into U-Net.	Enhanced gradient flow and reduced vanishing gradient issues.	Good performance in large-scale and high-resolution images.	Struggles with smaller regions and overlapping tumors.
Proposed Model	Modified U-Net with spatial attention.	Achieved superior boundary delineation, particularly in low-contrast and overlapping regions.	Lightweight design, robust boundary accuracy.	Sensitive to extreme noise; requires preprocessing consistency.

**Table 2:** The downsampling layers of the proposed model.

Downsampling ↓ (down sampling)
ResBlock_1
Conv2d [3 × 3, f_in = 3, f_out = 16]
ReLU(max(0,x))
Conv2d [3 × 3, f_in = 16, f_out = 16]
ReLU(max(0,x))
Conv2d [3 × 3, f_in = 16, f_out = 16]
Spatial Attention Block_1
Concat(AvgPooling, MaxPooling)
Conv2d [1 × 1, f_in = 32, f_out = 32]
Sigmoid($\frac{1}{1+e^{-x}}$)
Conv2d [1 × 1, f_in = 16, f_out = 32]
MaxPooling
Multiplication
ResBlock_2
Conv2d [3 × 3, f_in = 32, f_out = 32]
ReLU(max(0,x))
Conv2d [3 × 3, f_in = 32, f_out = 32]
ReLU(max(0,x))
Conv2d [3 × 3, f_in = 32, f_out = 32]
MaxPooling
ResBlock_3
Conv2d [3 × 3, f_in = 32, f_out = 64]
ReLU(max(0,x))
Conv2d [3 × 3, f_in = 64, f_out = 64]
ReLU(max(0,x))
Conv2d [3 × 3, f_in = 64, f_out = 64]
Spatial Attention Block_2
Concat(AvgPooling, MaxPooling)
Conv2d [1 × 1, f_in = 64, f_out = 128]
Sigmoid($\frac{1}{1+e^{-x}}$)
Conv2d [1 × 1, f_in = 64, f_out = 128]
MaxPooling
Multiplication
ResBlock_4
Conv2d [3 × 3, f_in = 128, f_out = 128]
ReLU(max(0,x))
Conv2d [3 × 3, f_in = 128, f_out = 128]
ReLU(max(0,x))
Conv2d [3 × 3, f_in = 128, f_out = 128]
MaxPooling
ResBlock_5
Conv2d [3 × 3, f_in = 128, f_out = 256]
ReLU(max(0,x))
Conv2d [3 × 3, f_in = 256, f_out = 256]
ReLU(max(0,x))
Conv2d [3 × 3, f_in = 256, f_out = 256]

**Table 3:** The upsampling layers of the proposed model.

Upsampling ↑ (up sampling)
ResBlock_6
Upsample(Concat), 2)
Conv2d [3 × 3, f_in = 256, f_out = 128]
REL(max(0,x))
Conv2d [3 × 3, f_in = 128, f_out = 128]
ReLU(max(0,x))
Conv2d [3 × 3, f_in = 128, f_out = 128]
ResBlock_7
Upsample(Concat),4)
Conv2d [3 × 3, f_in = 128, f_out = 64]
ReLU(max(0,x))
Conv2d [3 × 3, f_in = 64, f_out = 64]
ReLU(max(0,x))
Conv2d [3 × 3, f_in = 64, f_out = 64]
ResBlock_8
Upsample(Concat),2)
Conv2d [3 × 3, f_in = 64, f_out = 32]
ReLU(max(0,x))
Conv2d [3 × 3, f_in = 32, f_out = 32]
ReLU(max(0,x))
Conv2d [3 × 3, f_in = 32, f_out = 32]
ResBlock_9
Upsample(Concat),4)
Conv2d [3 × 3, f_in = 32, f_out = 16]
ReLU(max(0,x))
Conv2d [3 × 3, f_in = 16, f_out = 16]
ReLU(max(0,x))
Conv2d [3 × 3, f_in = 16, f_out = 16]
Conv2d [3 × 3, f_in = 16, f_out = 3]

**Table 4:** Comparison results with 95% confidence intervals.

Model	Architecture	Accuracy (%)	Precision (%)	F1-Score (%)	AUC	DSC (%)	Recall (%)
V-Net [13]	Volumetric U-Net	81.0 ± 1.6	84.0 ± 1.5	82.76 ± 1.6	0.86 ± 1.4	88.0 ± 1.4	90.0 ± 1.5
DeepLab V3+ [25]	Atrous convolutions	83.0 ± 1.4	86.0 ± 1.2	84.87 ± 1.4	0.88 ± 1.2	91.0 ± 1.4	94.0 ± 1.4
ResUNet-a [14]	U-Net with residual connections	85.0 ± 1.8	87.0 ± 1.6	83.29 ± 1.6	0.89 ± 1.6	90.0 ± 1.5	92.0 ± 1.6
nnU-Net [26]	Self-adapting framework based on U-Net	87.0 ± 1.0	89.0 ± 1.0	85.32 ± 1.2	0.91 ± 1.2	92.0 ± 1.0	93.0 ± 1.2
Baseline [4]	Standard U-Net	85.0 ± 1.2	86.0 ± 1.0	84.69 ± 1.1	0.88 ± 1.1	90.0 ± 1.2	92.0 ± 1.2
Proposed Model	Modified U-Net with spatial attention	89.0 ± 0.8	91.0 ± 0.5	88.67 ± 0.4	0.94 ± 0.4	93.0 ± 0.5	95.0 ± 0.4

**Table 5:** Comparison with SOTA models including Hausdorff distance.

Model	DSC	Precision	Recall	AUC	Hausdorff Distance (95%)
V-Net [13]	0.88	0.84	0.90	0.86	9.12
DeepLab V3+ [25]	0.91	0.86	0.94	0.88	7.89
ResUNet-a [14]	0.90	0.87	0.92	0.89	8.45
nnU-Net [26]	0.92	0.89	0.93	0.91	6.75
Proposed Model	0.93	0.91	0.95	0.94	5.42

**Table 6:** Results of the BraTS dataset.

Model	DSC	Precision	Recall	Hausdorff Distance (95%)
Baseline U-Net	0.87	0.85	0.88	8.74
nnU-Net	0.90	0.89	0.91	6.80
Proposed Model	0.91	0.90	0.92	6.02

**Table 7:** Robustness and reliability of the proposed model.

Dataset	Metric	Mean	Standard Deviation
Figshare	DSC	0.93	±0.004
	Precision	0.91	±0.005
	Recall	0.95	±0.003
	Hausdorff distance (95%)	5.42	±0.12
BraTS	DSC	0.91	±0.006
	Precision	0.90	±0.007
	Recall	0.92	±0.004
	Hausdorff distance (95%)	6.02	±0.15

**Table 8:** Identified bottlenecks, example scenarios, and proposed resolutions for the modified U-Net model.

Bottleneck	Description	Proposed Resolution
Low-Contrast Tumor Boundaries	The model struggles to delineate tumor edges with low contrast compared to the surrounding tissues.	1. Enhancing preprocessing with adaptive histogram equalization. 2. Fine-tuning the spatial attention mechanism.
Small Tumor Regions	Small tumors are partially detected or overlooked, resulting in fragmented segmentation.	1. Incorporating multi-scale feature extraction. 2. Using higher-resolution input images.
Complex Overlapping Tumors	Overlapping tumors are sometimes merged into a single region, reducing segmentation accuracy.	1. Adding postprocessing with connected component analysis. 2. Integrating an auxiliary classification head.

## Data Availability

All used datasets are available online with open access.
